# Primary neuroendocrine tumors of the ovary: Management and outcomes

**DOI:** 10.1002/cam4.4368

**Published:** 2021-11-12

**Authors:** Li Pang, Zhiqiang Guo

**Affiliations:** ^1^ Department of Obstetrics and Gynecology Shengjing Hospital of China Medical University Shenyang Liaoning China

**Keywords:** chemotherapy, clinical behavior, ovarian cancer, ovarian neuroendocrine tumor, SEER database

## Abstract

**Background:**

There is currently no recognized first‐line treatment strategy for ovarian neuroendocrine tumors (NETs). Furthermore, because of the low incidence of ovarian NETs, no studies have reported prognostic statistics derived from large samples. This retrospective study aimed to investigate the clinical behavior of ovarian NETs.

**Methods:**

The Surveillance, Epidemiology, and End Results database was used to identify women diagnosed with ovarian NETs from 2004 to 2015. Overall survival (OS), cancer‐specific survival (CSS), and independent prognostic factors for ovarian NETs were evaluated. The effects of different treatments on prognosis were also compared, as were OS and CSS rates for histological subtypes.

**Results:**

The 5‐year OS rates were 83.3%, 30.0%, 20.3%, and 9.8% for patients in stages I (*n* = 159), II (*n* = 23), III (*n* = 101), and IV (*n* = 148), respectively. The 5‐year CSS rates were 85.6%, 41.7%, 21.2%, and 9.8% for patients in stages I–IV, respectively. Age, American Joint Committee on Cancer (AJCC) stage, lymph node metastasis, treatment, and histological type were related to poor OS and CSS. In the early stage, the 5‐year OS and CSS rates were 97.03% and 96.90%, respectively. For patients in the advanced stage receiving comprehensive treatment (surgery + chemotherapy + radiotherapy), the 5‐year OS and CSS rates were 72.9% and 70.00%, respectively. When comparing low‐ and high‐grade neuroendocrine carcinoma, 5‐year OS rates were 93.96% and 7.01%, 5‐year CSS rates were 97.44% and 7.31%, 10‐year OS rates were 93.56% and 2.34%, and 10‐year CSS rates were 97.44% and 4.88%, respectively.

**Conclusion:**

Age, AJCC stage, treatment, and histological type are independent prognostic factors of ovarian NETs. OS and CSS are relatively good for early‐stage cases treated with surgery alone, whereas more comprehensive treatment is required to improve OS and CSS in the advanced stage.

## Graphical abstract

We assessed ovarian neuroendocrine tumors’ clinical behavior via the Surveillance, Epidemiology, and End Results database. Age, American Joint Committee on Cancer stage, lymph node metastasis, treatment, and histological type predicted poor overall and cancer‐specific survival. Prognosis was relatively good among patients with early‐stage disease treated with surgery alone. Comprehensive (surgery + chemotherapy + radiotherapy) treatment can improve prognosis in patients with advanced disease. Prognosis is better for low‐grade neuroendocrine carcinoma than for high‐grade neuroendocrine carcinoma.

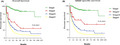

## 1 INTRODUCTION

Derived from neuroendocrine cells, neuroendocrine tumors (NETs) are aggressive diseases that often occur in the gastrointestinal tract, pancreas, and lungs. Cases occurring in other tissues and organs are rare, especially in the female reproductive tract.^1^ Ovarian NETs account for only 1%–2% of malignant ovarian tumors.^2^, ^3^ At present, these tumors can be roughly divided into carcinoid, atypical carcinoid (ACT), small‐cell carcinoma, and large‐cell neuroendocrine carcinoma (LCNEC) types.^3^ According to World Health Organization (WHO) regulations, non‐small‐cell neuroendocrine carcinoma (NSCNEC) is similar to LCNEC.^4^ Carcinoid and ACT are classified as low‐grade NETs, whereas the small‐cell carcinoma, LCNEC, and NSCNEC are classified as high‐grade neuroendocrine carcinomas.^4^ High‐grade neuroendocrine carcinomas are considered more aggressive than low‐grade NETs.^4^, ^5^, ^6^

Due to the low incidence of ovarian NETs, clinicians may be unaware of appropriate treatments for the disease and factors influencing patient prognosis, which may cause them to miss the window of opportunity for treatment. Furthermore, few cases of ovarian NETs have been reported in the literature, and such cases are often reported in small series only. Moreover, there is currently no recognized first‐line treatment strategy, and no studies have reported prognostic statistics derived from large samples.

The Surveillance, Epidemiology, and End Results (SEER) database is considered a powerful tool for identifying population characteristics and studying the long‐term prognosis of rare tumors.^7^ In the present study, we used the SEER database to extract relevant patient information and analyze the clinical behavior and prognostic factors of ovarian NETs. In addition, we systematically assessed the prognosis of ovarian NET and evaluated the effect of ovarian NET treatment on prognosis. Finally, we compared the data between low‐grade NETs and high‐grade neuroendocrine carcinomas.

## 2 MATERIALS AND METHODS

### 2.1 Eligibility criteria and data collection

This retrospective observational cohort study utilized the SEER database of the National Cancer Institute.^7^ The SEER program is the largest population‐based tumor registration system in the United States. It was launched in 1973 and has a history of more than 40 years of operation, covering approximately 34.6% of the U.S. population in the most recent update. It is considered a powerful tool for identifying population characteristics and studying the long‐term prognosis of rare tumors. Patients were identified using the SEER database, and those histologically diagnosed with ovarian NETs from 2004 to 2015 who met the following criteria were considered eligible: primary malignant tumor in the ovary (ICD‐O‐3/WHO 2008 site code), carcinoid tumor (8240/3), ACT (8249/3), large‐cell carcinoma (8012/3), LCNEC (8013/3), non‐small‐cell carcinoma (8046/3), and small‐cell carcinoma (8041/3). The exclusion criteria were as follows: diagnosis of benign or borderline tumors and cases in which the ovarian NET was not the first tumor.

Notably, the use of the SEER database has some limitations. For example, the SEER database does not include information regarding the number of chemotherapy cycles, specific chemotherapy regimens, or neoadjuvant chemotherapy regimens. In addition, the database does not specify the time or location of recurrence, nor does it mention the treatments used in such cases. Preoperative imaging data are also currently unavailable in the SEER database. Lastly, the SEER database lacks objective indicators of carcinoid involvement of the heart and does not distinguish between transabdominal and laparoscopic surgery.

SEER*Stat 8.3.8 software (https://seer.cancer.gov/data/) was used to generate case listings. The deidentified data in the SEER database are publicly available; thus, their use is exempt from review by the Shengjing Hospital of China Medical University Institutional Review Board. The same software was used to record patient information, including demographic characteristics, clinical pathology findings, and treatment parameters. Staging information was determined based on the American Joint Committee on Cancer (AJCC) staging system.

### 2.2 Clinical and demographic characteristics

Demographic data in this analysis included age at diagnosis (≤49, 50–69, and ≥70 years), AJCC stage (I, IA, IB, IC, II, IIA, IIB, IIC, III, IIIA, IIIB, IIIC, and IV), year of diagnosis (2004–2009 and 2010–2015), grade (well‐differentiated, moderately differentiated, poorly differentiated, and undifferentiated), tumor size (≤5.0 cm, >5.0 cm, and unknown), nodal metastasis (negative, positive, not examined, and unknown), sampled pelvic nodes (1–9, 10–19, ≥20, not examined, and unknown), distant metastasis (bone, brain, liver, lung, none, and unknown), and treatment (surgery alone, surgery + chemotherapy [CTX], surgery + concurrent chemoradiotherapy [CCRT], CTX alone, radiotherapy [RT] + CTX, RT alone, and no treatment). Follow‐up time after diagnosis, life status, and cause of death were collected from the database to evaluate survival due to disease (i.e., cancer‐specific survival [CSS] and overall survival [OS]).

### 2.3 Statistical analysis

Categorical data are presented as numbers and percentages (*N*, %). The chi‐square test or Fisher's exact test was used to compare the clinical and demographic characteristics of women with low‐grade NETs and high‐grade neuroendocrine carcinomas. Univariate and multivariate Cox regression analyses were used to determine independent predictors of OS and CSS in patients with ovarian NETs. OS and CSS were calculated using Kaplan–Meier curves, and the log‐rank test was used for comparison. All data were analyzed using SPSS software (version 23.0; SPSS). GraphPad Prism (8.3.0 GraphPad Software) was used to draw Kaplan–Meier survival curves, and *p* values < 0.05 were considered statistically significant.

## 3 RESULTS

### 3.1 Patients

A total of 431 cases of ovarian NETs in the SEER registry met the inclusion criteria. Table 1 summarizes the characteristics of patients with ovarian NETs, including age, year at diagnosis, AJCC stage, tumor size, grade, nodal metastasis, sampled pelvic nodes, distant metastasis, and treatment. There were 123 cases of low‐grade NETs (carcinoid: 118, ACT: 5), 254 cases of high‐grade neuroendocrine carcinomas (SCNEC: 124, LCNEC: 130), and 54 cases of NET (not classified). Table 1 shows the proportion of patients in each AJCC stage. Stage I disease was observed in 36.4% of patients, while the proportion of patients with poorly differentiated and undifferentiated types was greater by approximately 22.3%. The treatment strategy accounting for the largest proportion of patients was surgery alone (37.6%). The findings also indicated that more patients with stage I ovarian neuroendocrine cancer underwent separate surgical treatments, and that poorly differentiated and undifferentiated cases were most common in this group.

**TABLE 1:** Patient characteristics of neuroendocrine tumors of the ovary

Subject	*n* = 431	(%)
Hystological type		
LGNET	123	28.5
Carcinoid	118	27.4
ACT	5	1.1
HGNEC	254	58.9
SCNEC	124	28.8
LCNEC	130	30.1
NET, not elsewhere classified	54	12.6
Age (years)		
≤49	173	40.1
50–69	139	32.3
≥70	119	27.6
Year at diagnosis		
2004–2009	154	35.7
2010–2015	277	64.3
AJCC stage		
I		
IA	123	28.5
IB	2	0.5
IC	27	6.3
INOS	7	1.6
II		
IIA	5	1.2
IIB	12	2.8
IIC	6	1.4
III		
IIIA	3	0.7
IIIB	3	0.7
IIIC	61	14.2
IIINOS	34	7.8
IV	148	34.3
Grade		
Well differentiated	35	8.1
Moderately differentiated	17	3.9
Poorly differentiated	44	10.2
Undifferentiated differentiated	52	12.1
Unknown	283	65.7
Tumor size (cm)		
≤5.0	79	18.3
>5.0	207	48
Unknown	145	33.7
Nodal metastasis		
Negative	73	16.9
Positive	50	11.6
Not examined	301	69.8
Unknown	7	1.7
Sampled pelvic nodes		
1–9	59	13.7
10–19	34	7.9
≥20	30	7
Not examined	301	69.8
Unknown	7	1.6
Distant metastasis		
Bone	10	2.3
Brain	2	0.5
Liver	33	7.7
Lung	12	2.8
No	231	53.5
Unknown	143	33.2
Treatment		
Surgery alone	162	37.6
Surgery + CTX	116	26.9
Surgery + CCRT	17	3.9
CTX alone	62	14.4
RT + CTX	8	1.9
RT alone	4	0.9
No treatment	62	14.4

Note

*n*: Number.

Abbreviations: ACT, atypucal carcinoid tumor; AJCC, American Joint Commission on Cancer; CCRT, concurrent chemoradiation; CTX, chemotherapy; HGNEC, high‐grade neuroendocrine carcinoma; LCNEC, large cell neuroendocrine carcinoma; LGNET, low‐grade neuroendocrine tumor; NET, neuroendocrine tumor; NOS, not otherwise specified; RT, radiation; SCNEC, small‐cell neuroendocrine carcinoma.

The treatment for each stage is summarized in Table S1. Among the 431 included patients, 162 underwent surgery alone. Among the 159 patients in stage I, surgery alone was the primary treatment in 125 patients (78.6%). Most of the 116 patients who underwent surgery + CTX (69.8%) were in the advanced stage, with 46 in stage III and 35 in stage IV. Seventeen patients underwent surgery + CCRT, whereas 62 patients received CTX only, and most of these patients (91.9%) were also in the advanced stage (stage III: 18, stage IV: 39). Only eight patients in stage IV received RT + CTX. Four patients received RT only, including two patients in stage III and two patients in stage IV (Table S1).

### 3.2 Survival curves

**FIGURE 1:**
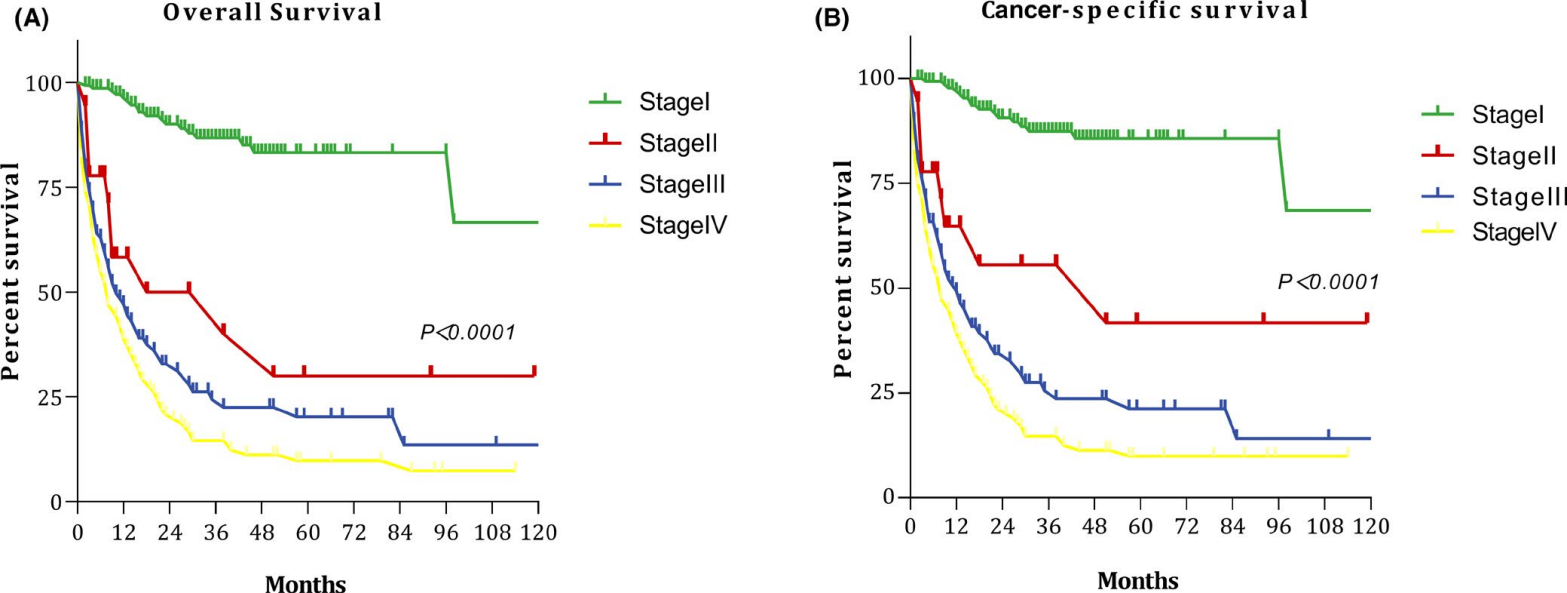
Survival curves at each stage: (A) overall survival; and (B) cancer‐specific survival

Figure 1 shows the OS and CSS curves for patients in each stage. The 5‐year OS rates for patients with stage I, II, III, and IV disease were 83.3%, 30.0%, 20.3%, and 9.8%, respectively. The 5‐year CSS rates for patients with stages I, II, III, and IV were 85.6%, 41.7%, 21.2%, and 9.8%, respectively. Figure S1 shows the survival curves for patients in different stages. The figure shows that survival time still gradually decreased as the stage increased.

**FIGURE 2:**
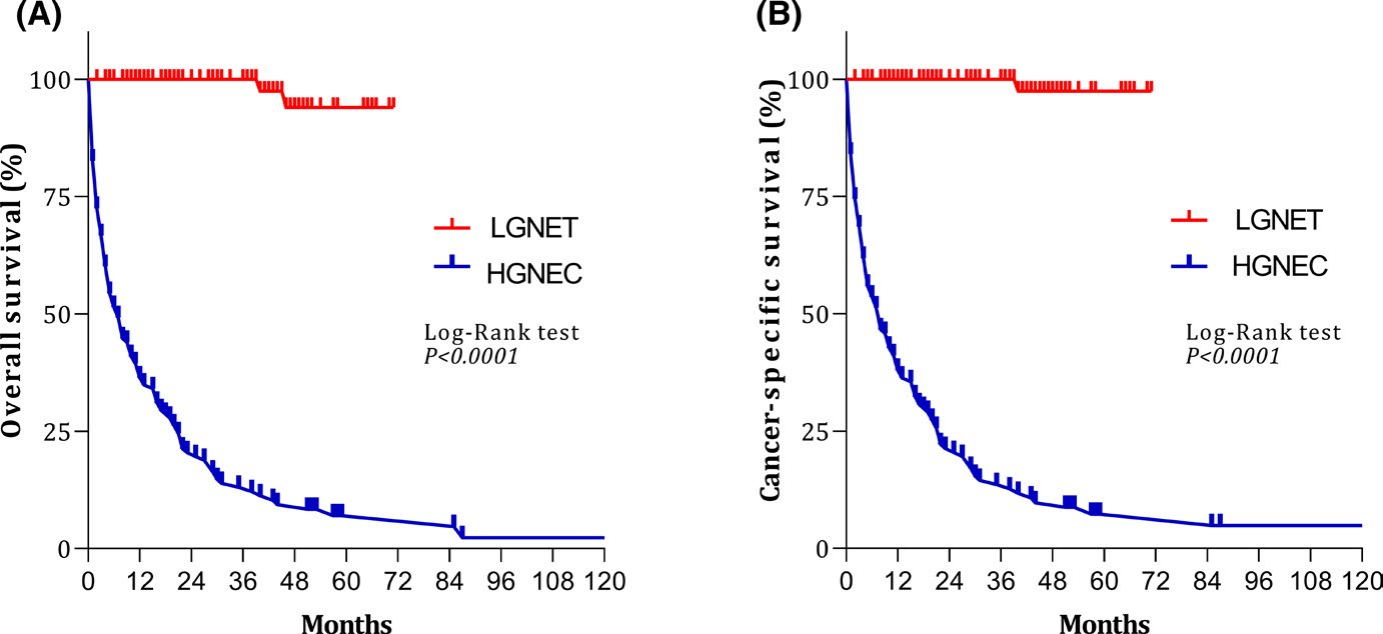
Survival curves for patients with low‐grade neuroendocrine tumors (LGNET) and high‐grade neuroendocrine carcinoma (HGNEC): (A) overall survival; and (B) cancer‐specific survival

Figure 2 shows the OS and CSS curves of patients with low‐grade NETs and high‐grade neuroendocrine carcinomas. The 5‐year OS rates for low‐grade NETs and high‐grade neuroendocrine carcinomas were 93.96% and 7.01%, respectively. The 5‐year CSS rates for low‐grade NETs and high‐grade neuroendocrine carcinomas were 97.43% and 7.31%, respectively.

### 3.3 Prognostic factors

To determine factors influencing prognosis in patients with ovarian NET, we selected histological type (low‐grade NETs vs. high‐grade neuroendocrine carcinomas), age, AJCC stage, tumor size, nodal metastasis, distant metastasis, and treatment as variables for univariate and multivariate analyses (Table 2). Multivariate analysis revealed that age, AJCC stage, nodal metastasis, treatment, and histological type were associated with poor OS and CSS. Tumor size and distant metastasis were not identified as prognostic factors.

**TABLE 2:** Prognostic factors for NETs of the ovary

Subject characteristics	Overall survival				Cancer‐specific survival			
	Univariate		Multivariate		Univariate		Multivariate	
	HR (95% CI)	*p* value	HR (95% CI)	*p* value	HR (95% CI)	*p* value	HR (95% CI)	*p* value
Age (years)								
≤49	1		1		1		1	
50–69	0.936 (0.669–1.308)	0.698	0.525 (0.345–0.798)	0.003	0.903 (0.644–1.267)	0.555	0.493 (0.323–0.752)	0.001
≥70	2.387 (1.766–3.228)	＜0.001	0.660 (0.439–0.991)	0.045	2.156 (1.582–2.937)	＜0.001	0.572 (0.378–0.867)	0.008
AJCC stage								
I	1		1		1		1	
II	6.221 (2.956–13.091)	＜0.001	2.718 (1.168–6.323)	0.020	5.768 (2.568–12.956)	＜0.001	2.753 (1.134–6.684)	0.025
III	10.241 (6.175–16.983)	＜0.001	3.025 (1.600–5.718)	0.001	10.715 (6.292–18.247)	＜0.001	3.403 (1.767–6.552)	＜0.001
IV	13.94 (8.570–22.690)	＜0.001	3.441 (1.745–6.784)	＜0.001	15.432 (9.255–25.732)	＜0.001	4.039 (2.006–8.131)	＜0.001
Tumor size (cm)								
≤5.0	1		1		1		1	
>5.0	3.505 (2.009–6.117)	＜0.001	1.650 (0.858–3.173)	0.133	3.612 (2.029–6.431)	＜0.001	1.650 (0.836–3.254)	0.149
Unknown	6.347 (3.631–11.096)	＜0.001	1.615 (0.848–3.076)	0.145	6.518 (3.654–11.626)	＜0.001	1.641 (0.841–3.203)	0.147
Nodal metastasis								
Negative	1		1		1		1	
Positive	3.044 (1.795–5.160)	＜0.001	1.407 (0.732–2.704)	0.306	3.225 (1.870–5.562)	＜0.001	1.327 (0.683–2.578)	0.403
Not examined	2.609 (1.694–4.019)	＜0.001	1.884 (1.094–3.244)	0.022	2.679 (1.710–4.197)	＜0.001	1.873 (1.079–3.253)	0.026
Unknown	9.910 (4.210–23.329)	＜0.001	3.589 (1.304–9.883)	0.013	10.790 (4.544–25.623)	＜0.001	3.585 (1.293–9.937)	0.014
Distant metastasis								
Yes	1		1		1		1	
No	0.203 (0.132–0.312)	＜0.001	1.020 (0.568–1.831)	0.948	0.186 (0.120–0.288)	＜0.001	0.976 (0.540–1.764)	0.937
Unknown	0.856 (0.582–1.261)	0.432	1.080 (0.651–1.794)	0.765	0.823 (0.558–1.214)	0.326	1.014 (0.609–1.689)	0.957
Histological type								
LGNET	1		1		1		1	
HGNEC	36.849 (13.681–99.254)	＜0.001	26.270 (8.173–84.438)	＜0.001	47.582 (15.197–148.98)	＜0.001	34.525 (9.385–127.015)	＜0.001
Treatment								
Surgery alone	1		1		1		1	
Surgery + CTX	3.462 (2.258–5.308)	＜0.001	0.384 (0.229–0.644)	＜0.001	3.529 (2.288–5.444)	＜0.001	0.357 (0.212–0.602)	＜0.001
Surgery + CCRT	0.933 (0.328–2.652)	0.898	0.081 (0.023–0.280)	＜0.001	0.724 (0.221–2.378)	0.595	0.073 (0.021–0.256)	＜0.001
CTX alone	8.128 (5.18512.743)	＜0.001	0.683 (0.370–1.258)	0.221	8.235 (5.220–12.993)	＜0.001	0.634 (0.340–1.180)	0.150
RT + CTX	6.992 (3.060–15.979)	＜0.001	0.607 (0.220–1.669)	0.333	7.233 (3.156–16.573)	＜0.001	0.549 (0.198–1.521)	0.249
RT alone	9.084 (2.744–30.082)	＜0.001	1.385 (0.398–4.819)	0.608	9.457 (2.849–31.387)	＜0.001	1.419 (0.407–4.947)	0.583
No treatment	17.578 (11.151–27.711)	＜0.001	2.066 (1.105–3.864)	0.023	16.302 (10.196–26.063)	＜0.001	1.787 (0.941–3.394)	0.076

Abbreviations: AJCC, American Joint Commission on Cancer; CCRT, concurrent chemoradiation; CTX, chemotherapy; CI, confidence interval; HGNEC, high‐grade neuroendocrine carcinoma; HR, hazard ratio; LGNET, low‐grade neuroendocrine tumor; NET, neuroendocrine tumor; RT, radiation.

### 3.4 Treatment and prognosis

The main surgical treatments included unilateral salpingo‐oophorectomy, bilateral salpingo‐oophorectomy, total hysterectomy + bilateral salpingo‐oophorectomy + pelvic lymph node dissection, ovarian cancer cytoreductive surgery, and adjuvant treatments, such as chemotherapy and radiotherapy. Only 16 of the 431 patients underwent resection of the adnexa of the affected side, which preserved the endocrine function. Among them, 14 had ovarian carcinoid, one had large‐cell carcinoma, and one had an ACT.

**FIGURE 3:**
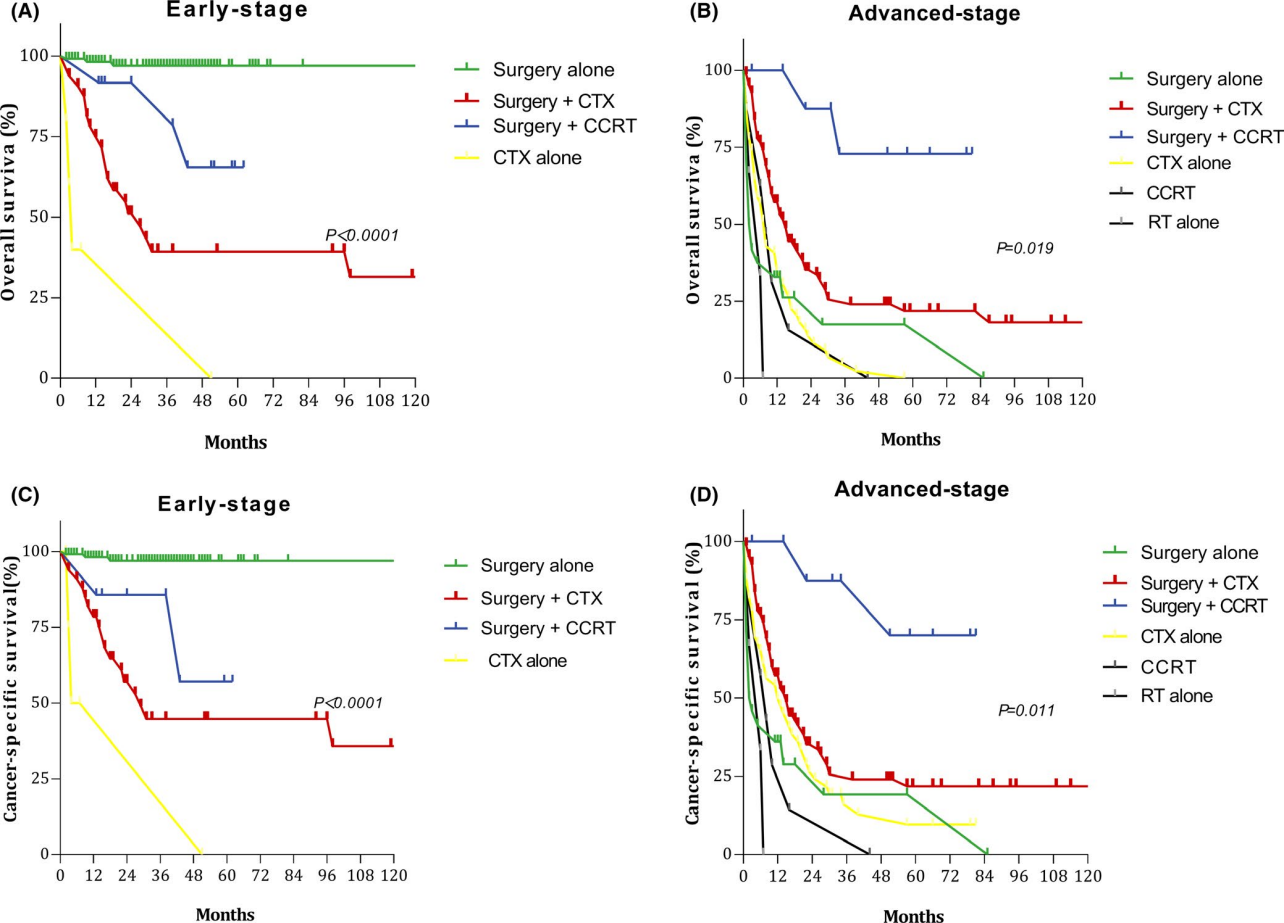
Survival curves for patients with early‐ and advanced‐stage disease for different treatment regimens: (A) overall survival (OS) in the early stage; (B) OS in the advanced stage; (C) cancer‐specific survival (CSS) in the early stage; and (D) CSS in the advanced stage

In the early stages (I–II), 132 patients received surgical treatment only. Their 5‐year OS and CSS rates were 97.03% and 96.90%, respectively. Thirty‐five patients underwent surgery and CCRT. Five‐year OS and CSS rates in these patients were 65.5% and 57.1%, respectively. Ten patients in the advanced stage received surgery + CTX + RT, and the 5‐year OS and CSS rates were 72.9% and 70.0%, respectively, in patients receiving such comprehensive treatment. However, the 5‐year OS and CSS rates were only 17.5% and 19.3% after surgery alone (Table 3). Among patients with early‐stage disease, 159 were in stage I, whereas 23 were in stage II. These findings indicate that OS and CSS were relatively good for early‐stage cases treated with surgery alone, whereas more comprehensive treatment (surgery + CTX + RT) was required to improve OS and CSS in the advanced stage (Figure 3).

**TABLE 3:** Five‐year OS and CSS according to stage and treatment in patients with ovarian neuroendocrine tumors

Treatments	*N*	5‐year OS (%)	*p*	5‐year CSS (%)	*p*
Stage I–II					
Surgery alone	132	97.03	<0.001	96.90	<0.001
Surgery + CTX	35	39.30		44.80	
Surgery + CCRT	7	65.50		57.10	
CTX alone	5	0		0	
CCRT	0	0		0	
Stage III–IV					
Surgery alone	30	17.50	0.019	19.30	0.011
Surgery + CTX	81	21.80		21.80	
Surgery + CCRT	10	72.90		70.00	
CTX alone	57	0		9.60	
CCRT	8	0		0	
RT alone	4	0		0	

Abbreviations: CCRT, concurrent chemoradiotherapy; CSS, cancer‐specific survival; CTX, chemotherapy; OS, overall survival; RT, radiotherapy.

### 3.5 Comparison between histological subtypes

The low‐grade NET (*n* = 123) and high‐grade neuroendocrine carcinoma groups (*n* = 254) were compared in terms of patient age, AJCC stage, grade, tumor size, nodal metastasis, sampled pelvic nodes, distant metastasis, treatment, and survival rate (Table 4). Most patients with low‐grade NETs were in stage I (92.7% low‐grade vs. 14.2% high‐grade neuroendocrine carcinomas), whereas most patients with high‐grade neuroendocrine carcinomas were in stage III or IV (5.7% low‐grade vs. 79.9% high‐grade neuroendocrine carcinomas). In addition, the low‐grade NET was associated with lower rates of distant and nodal metastasis than high‐grade neuroendocrine carcinoma (*p* < 0.001). Most patients with low‐grade NETs were treated with surgery alone (94.35%), whereas the most common treatment among patients with high‐grade neuroendocrine carcinomas was surgery + CTX (37.8%). The 5‐ and 10‐year OS and CSS rates were much higher for low‐grade NETs than for high‐grade neuroendocrine carcinomas.

**TABLE 4:** Comparison between LGNET and HGNEC

Subject characteristics	LGNET	HGNEC	*p* value
	*n* (%)	*n* (%)	
	123 (32.6)	254 (67.4)	
Age			<0.001
≤49	58 (47.1)	98 (38.6)	
50–69	54 (43.9)	67 (26.3)	
≥70	11 (9.0)	89 (25.1)	
AJCC stage			<0.001
I	114 (92.7)	36 (14.2)	
II	2 (1.6)	15 (5.9)	
III	3 (2.4)	81 (31.9)	
IV	4 (3.3)	122 (48.0)	
Grade			
Well/moderately differentiated	42 (34.1)	0 (0.0)	<0.001
Poorly/undifferentiated differentiated	2 (1.6)	111 (43.7)	
Unknown	79 (64.3)	143 (56.3)	
Tumor size (cm)			<0.001
≤5.0	52 (42.3)	18 (7.1)	
>5.0	44 (35.8)	131 (51.6)	
Unknown	27 (21.9)	105 (41.3)	
Nodal metastasis			
Negative	18 (14.6)	47 (18.5)	
Positive	1 (0.8)	37 (14.6)	
Not examined	102 (83.0)	165 (65.0)	
Unknown	2 (1.6)	5 (1.9)	
Sampled pelvic nodes			0.003
1–9	8 (6.5)	43 (16.9)	
10–19	9 (7.3)	19 (7.5)	
≥20	3 (2.4)	22 (8.7)	
Not examined	102 (83.0)	165 (65.0)	
Unknown	1 (0.8)	5 (1.9)	
Distant metastasis			<0.001
Yes	3 (2.4)	24 (9.4)	
No	120 (97.6)	76 (30.0)	
Unknown	0 (0)	154 (60.6)	
Treatment			<0.001
Surgery alone	116 (94.3)	31 (12.2)	
Surgery + CTX	2 (1.6)	96 (37.8)	
Surgery + CCRT	0 (0)	15 (5.9)	
CTX alone	2 (1.6)	54 (21.3)	
RT + CTX	1 (0.8)	6 (2.4)	
RT alone	0 (0)	3 (1.2)	
No treatment	2 (1.6)	49 (19.2)	
Survival rate			
5 year‐OS	93.56%	7.01%	
5 year‐CCS	97.44%	7.31%	
10 year‐OS	93.56%	2.34%	
10 year‐CCS	97.44%	4.88%	

Note

*n* (%): Number (%).

Abbreviations: AJCC, American Joint Commission on Cancer; CCRT, concurrent chemoradiation; CCS, cancer‐specific survival; CTX, chemotherapy; HGNEC, high‐grade neuroendocrine carcinoma; LGNET, low‐grade neuroendocrine tumor; OS, overall survival; RT, radiation.

## 4 DISCUSSION

In this study, we investigated the treatment strategies, prognostic factors, and outcomes in the largest cohort of patients with ovarian NETs to date. Our key finding is that age, AJCC stage, treatment method, and histological type were identified as independent prognostic factors for ovarian NET. In addition, our analysis revealed that OS and CSS are relatively good for early‐stage cases treated with surgery alone, whereas more comprehensive treatment is required to improve OS and CSS for cases in the advanced stage. Prognosis was also poorer for patients with high‐grade neuroendocrine carcinomas than for those with low‐grade NETs.

Given the rare incidence of ovarian NETs, most of the published literature consists of case reports and small case series.^8^, ^9^, ^10^, ^11^ Moreover, this disease entity encompasses several tumor types, with the largest single series reporting 58 cases of ovarian large‐cell carcinoma.^12^ The median survival time for patients with LCNEC of the ovary is 10 months. In their analysis of 329 cases, Soga et al.^13^ divided patients into two carcinoid groups based on the presence or absence of a related dermoid. The authors reported 5‐year survival rates of 84%–93.7% for these two groups. In addition, there are 11 case reports,^14^ all of which involved patients who underwent primary surgery. Five of these patients received adjuvant CTX with platinum therapy. The median OS time among 11 patients was 20 months. Preda et al.^8^ reported 34 cases of primary ovarian NETs. The average follow‐up time was 4.5 years, 22 out of 34 patients showed no signs of recurrence, and the disease remained static; 12 out of 34 patients had metastases. Five patients received somatostatin analogs or chemotherapy, and eight patients (23.5%) died of the disease. However, no previous large‐sample studies have investigated ovarian NETs, highlighting the practical clinical significance of the current study.

Establishing a diagnosis of ovarian NET can be challenging. Immunohistochemistry can help identify these tumors because they express markers of neuroendocrine differentiation, such as neuron‐specific enolase, synaptophysin, chromogranin CD56, vimentin, and epithelial membrane antigen.^4^, ^15^ The diagnosis can be confirmed by immunohistochemistry using one or more standard neuroendocrine markers. At present, SMARCA4^16^, ^17^, ^18^ is recognized as a biological marker of hypercalcemic‐type small‐cell carcinoma. Unfortunately, there are currently no candidate drugs for targeted therapy in these patients.

Primary ovarian NET can develop in any form; it is related to teratomas and originates from endocrine cells, whether ovarian or teratoma. Such NETs are divided into several groups according to their histopathological type: island, trabecular, mucinous (goblet), and stromal. However, patients usually present with two or more types.^19^, ^20^ Because they usually contain teratoma components, primary ovarian NETs are considered “specialized teratomas.” As the SEER database does not include molecular or histopathological data, we are unable to identify patients with de novo or teratoma‐related ovarian carcinoids.

Reed^21^ published a comprehensive review that describes a significant relationship between primary ovarian carcinoids and ovarian teratoma. The patients in their study were mostly in stage I and had non‐specific symptoms that could not be distinguished from ordinary epithelial ovarian cancer before surgery. The long‐term survival rate in these patients after surgery was >90%. However, such cases are different because they can be accompanied by cancer syndromes without metastatic disease. This is because the ovarian vein can drain directly into the inferior vena cava, which can lead to carcinoid syndrome and even occasional carcinoid heart disease, which may in turn be accompanied by right‐sided heart failure. Echocardiography is recommended to assess the presence of carcinoid heart disease. Measurement of N‐terminal pro‐B‐type natriuretic peptide (NT‐proBNP) can also be regarded as a screening test for carcinoid heart disease when carcinoid symptoms are present. Preda et al.^8^ reported 34 cases of primary ovarian NETs, including two cases (6%) of cardiac involvement. In 2000, Soga et al.^13^ noted that 6% of all patients and 25% of those with carcinoid symptoms exhibited cardiac involvement. Pelvic magnetic resonance imaging (MRI) can be considered the ideal local imaging technique, as it can aid surgeons in evaluating the feasibility of the operation. Fluorodeoxyglucose positron emission tomography (FDG‐PET)/computed tomography (CT) may be the best imaging technique for detecting metastasis. However, due to the high cost of PET/CT, a whole‐body CT scan is preferred when PET/CT is not available.^20^

Our multivariate analysis revealed that treatment options were related to prognosis. Patients with early‐stage ovarian NETs experienced good survival outcomes after surgery alone. The 5‐year OS and CSS rates were as high as 97.03% and 96.90%, respectively. For patients with advanced‐stage disease, comprehensive treatment (surgery + CTX + RT) was associated with improved OS and CSS. Ovarian carcinoids were also often small and unilateral tumors were confined to the ovaries. Unilateral salpingo‐oophorectomy also appears to be associated with high recovery rates,^20^ although other gastrointestinal metastases must be excluded. For patients with metastatic disease,^22^ it is recommended to conduct a thorough evaluation using MRI, or 68 Ga‐octreotate PET scanning to rule out the presence of other major sources (such as the gastrointestinal tract),^23^ and to ensure prompt initiation of appropriate treatment. Some scholars^1^ believe that somatostatin analogs (such as lanreotide and octreotide) should be considered if carcinoids are diagnosed, and that somatostatin analogs should be used before and during tumor resection to prevent carcinoid crisis complications.^24^ In a study by Nasioudis et al,^22^the authors were unable to verify that CTX can prolong survival time in patients with stages II–IV carcinoids, although another study noted that the Ki‐67 proliferation index may aid in the selection of patients likely to benefit from CTX.^25^ However, reports on ACT are rare. Indeed, there were only five cases in our study. Since ACT is an extremely rare disease, information regarding its biological behavior is scarce.^26^ Our results indicated that treatments were carried out in accordance with the carcinoid regimen and that large‐ and small‐cell carcinomas of the ovary were uncommon and classified as high‐grade neuroendocrine carcinomas. The literature reports that prognosis is poor, and that progression is rapid among these patients. Even when discovered early, the probability of metastasis or recurrence remains high.^12^, ^15^, ^20^, ^27^, ^28^, ^29^, ^30^ Thus, CTX, RT, or adjuvant treatment is recommended even in the early stage. Harrison et al.^31^ reported that the combination of RT and CTX provides the best chance of long‐term survival for patients with high‐grade neuroendocrine carcinomas. However, even with comprehensive treatment, the prevalence of relapse within 2 years remains high among patients with advanced‐stage disease.^27^, ^28^, ^32^

Since ovarian NETs are rare, there are no clear recommendations regarding the surgical treatment of ovarian carcinoid or ovarian NET. For ovarian carcinoids, fertility‐preserving surgery is allowed because these tumors are usually unilateral and have a good prognosis. Moreover, information regarding the safety of conservative treatment designed to preserve fertility in young women is very limited. However, if the patient recognizes the risk, fertility‐preserving surgery can be considered in the early stage.^1^, ^6^ As there were only 16 cases of fertility‐preserving surgery in our analysis, further studies are required to explore the safety of the procedure.

Due to the rarity of the disease and the lack of systematic population‐based research or registration data, treatment strategies for ovarian NETs have not been standardized. It is relatively unlikely that the tumor will be identified as NET prior to surgery; therefore, treatment will depend on surgical findings. In addition, most adjuvant treatments are based on the treatment plan for lung NETs, which comprise a combination of surgical resection and postoperative supplementation with platinum‐based CTX. The most commonly used drugs include cisplatin, carboplatin, doxorubicin, epirubicin, and etoposide.^27^, ^32^ Even in patients with relapse, the current treatment plan is still a comprehensive treatment based on platinum CTX, and postoperative adjuvant CTX is more common in patients with ovarian epithelial tumors, with relatively fewer patients receiving adjuvant RT. Surgery combined with RT and CTX plays an important role in patient prognosis, especially for those with advanced ovarian NETs. Taken together, the available evidence highlights the need to develop novel drug treatments, such as molecular targeting strategies. Pembrolizumab has been used in patients with recurrent cervical and vulvar NETs and has shown promise in phase II clinical trials.^33^ Therefore, clinical trials investigating the use of pembrolizumab for ovarian NETs are warranted.

We compared the characteristics of patients with different histological subtypes of ovarian NETs (low‐grade NETs vs. high‐grade neuroendocrine carcinomas). Low‐grade NETs were milder, more frequently presented as stage I disease, more often involved tumors with diameters <5 cm, and were associated with lower rates of lymph node positivity and distant metastasis than high‐grade neuroendocrine carcinomas. Among patients with low‐grade NETs, 94.3% were treated with surgery alone, and the 5‐year survival rate was as high as 93.56%. These findings indicate that prognosis is relatively better among patients with ovarian low‐grade NETs than among those with high‐grade neuroendocrine carcinomas.

The present study has some limitations. Importantly, the SEER database does not include information regarding the number of chemotherapy cycles, specific chemotherapy regimens, or neoadjuvant chemotherapy regimens. In addition, the time and location of recurrence are not specified, nor does it mention the treatments used in such cases, and the SEER database does not currently provide preoperative imaging data. Nonetheless, the use of the SEER database was also the major strength of our study, as it provides a large amount of data regarding cases of ovarian NETs over the past 12 years. To our knowledge, our study is the largest investigation of ovarian NETs conducted to date. Moreover, our data provide valuable clinical information regarding different treatment regimens and related prognostic information for patients with ovarian NETs.

In conclusion, the current research reveals that age, AJCC stage, treatment method, and histological type are independent prognostic factors for ovarian NETs. Moreover, our results indicate that OS and CSS are relatively good for early‐stage cases treated with surgery alone, whereas a more comprehensive treatment strategy involving surgery, CTX, and RT is required to improve OS and CSS in the advanced stage. Future studies should focus on the development of individualized treatment strategies to prolong survival time in patients with ovarian NETs.

## ETHICAL APPROVAL STATEMENT

The Shengjing Hospital of China Medical University Institutional Review Board waived the requirement for approval, as data from a public database were used.

## CONFLICT OF INTEREST

The authors report no conflicts of interest related to this work.

## Supporting information

Fig S1

Table S1

## Data Availability

All data were extracted from the Surveillance, Epidemiology, and End Results (SEER) database released in November 2019. Qualified researchers may access information on cancer statistics through the website of the SEER database (https://seer.cancer.gov/).

## References

[1] Gardner GJ , Reidy‐Lagunes D , Gehrig PA . Neuroendocrine tumors of the gynecologic tract: a Society of Gynecologic Oncology (SGO) clinical document. Gynecol Oncol. 2011;122(1):190‐198. 21621706 10.1016/j.ygyno.2011.04.011

[2] Guadagno EE , De Rosa G , Del Basso De Caro M . Neuroendocrine tumours in rare sites: differences in nomenclature and diagnostics‐a rare and ubiquitous histotype. J Clin Pathol. 2016;69(7):563‐574. 26915369 10.1136/jclinpath-2015-203551

[3] Yasuoka H , Tsujimoto M , Fujita S , et al. Monoclonality of composite large cell neuroendocrine carcinoma and mucinous epithelial tumor of the ovary: a case study. Int J Gynecol Pathol. 2009;28(1):55‐58. 19047907 10.1097/PGP.0b013e31817fb419

[4] Rouzbahman M , Clarke B . Neuroendocrine tumors of the gynecologic tract: select topics. Semin Diagn Pathol. 2013;30(3):224‐233. 24144291 10.1053/j.semdp.2013.06.007

[5] Eichhorn JH , Young RH . Neuroendocrine tumors of the genital tract. Am J Clin Pathol. 2001;115(suppl_1):S94‐S112. 11993694 10.1309/64CW-WKGK-49EF-BYD1

[6] Howitt BE , Kelly P , McCluggage WG . Pathology of neuroendocrine tumours of the female genital tract. Curr Oncol Rep. 2017;19(9):59. 28735441 10.1007/s11912-017-0617-2

[7] SEER*Stat Database: SEER 18 Regs Research Data . Surveillance, Epidemiology, and End Results (SEER) Program. Accessed November 15, 2019. www.seer.cancer.gov

[8] Preda VA , Chitoni M , Talbot D , Reed N , Grossman AB . Primary ovarian carcinoid: extensive clinical experience with an underrecognized uncommon entity. Int J Gynecol Cancer. 2018;28(3):466‐471. 29420361 10.1097/IGC.0000000000001215

[9] Burkeen G , Chauhan A , Agrawal R , et al. Gynecologic large cell neuroendocrine carcinoma: a review. Rare Tumors. 2020;12. doi:10.1177/2036361320968401 PMC7605029 33194158

[10] Peng X , Wang H . Primary pure large cell neuroendocrine carcinoma of the ovary: a rare case report and review of literature. Medicine (Baltimore). 2020;99(49):e22474. 33285672 10.1097/MD.0000000000022474 PMC7717854

[11] He Y , Zhao H , Li XM , Yin CH , Wu YM . A clinical analysis of small‐cell neuroendocrine carcinoma of the gynecologic tract: report of 20 cases. Arch Gynecol Obstet. 2019;299(2):543‐549. 30411160 10.1007/s00404-018-4960-9

[12] Yang X , Chen J , Dong R . Pathological features, clinical presentations and prognostic factors of ovarian large cell neuroendocrine carcinoma: a case report and review of published literature. J Ovarian Res. 2019;12(1):69. 31345245 10.1186/s13048-019-0543-z PMC6657379

[13] Soga J , Osaka M , Yakuwa Y . Carcinoids of the ovary: an analysis of 329 reported cases. J Exp Clin Cancer Res. 2000;19(3):271‐280. 11144518

[14] Sehouli J , Woopen H , Pavel M , et al. Neuroendocrine neoplasms of the ovary: a retrospective study of the North Eastern German Society of Gynecologic Oncology (NOGGO). Anticancer Res. 2016;36(3):1003‐1009. 26976990

[15] Vora M , Lacour RA , Black DR , Turbat‐Herrera EA , Gu X . Neuroendocrine tumors in the ovary: histogenesis, pathologic differentiation, and clinical presentation. Arch Gynecol Obstet. 2016;293(3):659‐665. 26306985 10.1007/s00404-015-3865-0

[16] Ramos P , Karnezia AN , Craig DW , et al. Small cell carcinoma of the ovary, hypercalcaemic type, displays frequent inactivating germline and somatic mutations in SMARCA4. Nat Genet. 2014;46(5):427‐429. 24658001 10.1038/ng.2928 PMC4332808

[17] Conlon N , Silva A , Guerra E , et al. Loss of SMARCA4 expression is both sensitive and specific for the diagnosis of small cell carcinoma of ovary, hypercalcemic type. Am J Surg Pathol. 2016;40(3):395‐403. 26645725 10.1097/PAS.0000000000000558 PMC4752399

[18] Witkowski L , Goudie C , Foulkes WD , McCluggage WG . Small‐cell carcinoma of the ovary of hypercalcemic type (malignant rhabdoid tumor of the ovary): a review with recent developments on pathogenesis. Surg Pathol Clin. 2016;9(2):215‐226. 27241105 10.1016/j.path.2016.01.005

[19] Talerman A . Germ cell tumour of the ovary. In: Kurman RJ , ed. Blaustein’s Pathology of the Female Genital Tract. 1st ed. Springer Verlag; 2002:967‐1033.

[20] Talerman A . Carcinoid tumors of the ovary. J Cancer Res Clin Oncol. 1984;107(2):125‐135. 6715397 10.1007/BF00399383 PMC12253509

[21] Reed NS . Neuroendocrine tumours of the gynecological tract. Curr Opin Oncol. 2016;28(5):412‐418. 27467970 10.1097/CCO.0000000000000321

[22] Nasioudis D , Frey MK , Chapman‐Davis E , Caputo TA , Holcomb K . Primary malignant ovarian carcinoid; management and outcomes. Gynecol Oncol. 2020;157(1):101‐105. 31959493 10.1016/j.ygyno.2020.01.002

[23] Klimstra DS , Modlin IR , Coppola D , Lloyd RV , Suster S . The pathologic classification of neuroendocrine tumors: a review of nomenclature, grading, and staging systems. Pancreas. 2010;39(6):707‐712. 20664470 10.1097/MPA.0b013e3181ec124e

[24] Oberg K , Kvols L , Caplin M , et al. Consensus report on the use of somatostatin analogs for the management of neuroendocrine tumors of the gastroenteropancreatic system. Ann Oncol. 2004;15(6):966‐973. 15151956 10.1093/annonc/mdh216

[25] Vilar E , Salazar R , Pérez‐García J , Cortes J , Oberg K , Tabernero J . Chemotherapy and role of the proliferation marker Ki‐67 in digestive neuroendocrine tumors. Endocr Relat Cancer. 2007;14(2):221‐232. 17639039 10.1677/ERC-06-0074

[26] Lou L , Zhou L , Wang W , Li H , Li Y . Atypical ovarian carcinoid tumor with widespread skeletal metastases: a case report of multiple endocrine neoplasia type 1 in a young woman. BMC Cancer. 2019;19(1):1107. 31727021 10.1186/s12885-019-6332-7 PMC6857273

[27] Nasioudis D , Chapman‐Davis E , Frey MK , Caputo TA , Witkin SS , Holcomb K . Small cell carcinoma of the ovary: a rare tumor with a poor prognosis. Int J Gynecol Cancer. 2018;28(5):932‐938. 29621125 10.1097/IGC.0000000000001243

[28] Safini F , Jouhadi H , El Attar H . Primary large cell neuroendocrine carcinoma of the ovary: a rare entity. Gulf J Oncol. 2021;1(35):82‐85. 33716217

[29] Zhu Y , Meng F , Fang H , Zhang Z , Wang L , Zheng W . Clinicopathologic characteristics and survival outcomes in neuroendocrine carcinoma of the ovary. Int J Gynecol Cancer. 2020;30(2):207‐212. 31796530 10.1136/ijgc-2019-000746

[30] Atienza‐Amores M , Guerini‐Rocco E , Soslow RA , Park KJ , Weigelt B . Small cell carcinoma of the gynecologic tract: a multifaceted spectrum of lesions. Gynecol Oncol. 2014;134(2):410‐418. 24875120 10.1016/j.ygyno.2014.05.017

[31] Harrison ML , Hoskins P , du Bois A , et al. Small cell of the ovary, hypercalcemic type: analysis of combined experience and recommendation for management. A GCIG study. Gynecol Oncol. 2006;100(2):233‐238. 16321429 10.1016/j.ygyno.2005.10.024

[32] Reed NS , Pautier P , Åvall‐Lundqvist E , et al. Gynecologic Cancer InterGroup (GCIG) consensus review for ovarian small cell cancers. Int J Gynecol Cancer. 2014;24(9 suppl 3):S30‐S34. 25341577 10.1097/IGC.0000000000000293

[33] Frumovitz M , Westin SN , Salvo G , et al. Phase II study of pembrolizumab efficacy and safety in women with recurrent small cell neuroendocrine carcinoma of the lower genital tract. Gynecol Oncol. 2020;158(3):570‐575. 32534809 10.1016/j.ygyno.2020.05.682 PMC7486997

