# Supplementary figures and images for: Primary neuroendocrine tumors of the ovary: Management and outcomes
Source: Cancer Med. 2021 Nov 12;10(23):8558–69. doi: 10.1002/cam4.4368 (PMC8633223; doi:10.1002/cam4.4368)

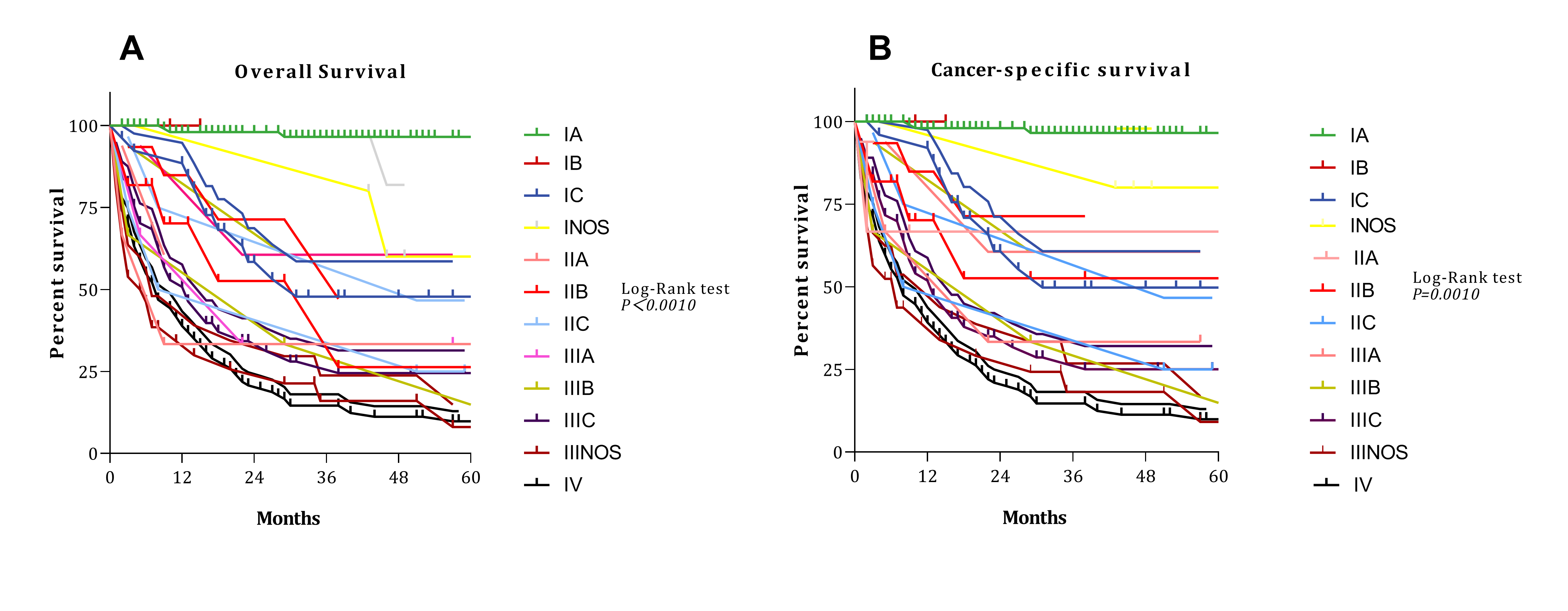

Supplement: Supplementary file 1 — Fig S1 [file CAM4-10-8558-s001.tif]
